# Interferon-gamma modulates articular chondrocyte and osteoblast metabolism through protein kinase R-independent and dependent mechanisms

**DOI:** 10.1016/j.bbrep.2022.101323

**Published:** 2022-09-07

**Authors:** S.J. Gilbert, E.J. Blain, D.J. Mason

**Affiliations:** Biomechanics & Bioengineering Centre Versus Arthritis, School of Biosciences, Cardiff University, CF10 3AX, UK

**Keywords:** Interferon-γ, PKR, Chondrocyte, Osteoblast, Inflammation, Osteoarthritis

## Abstract

Osteoarthritis (OA) affects multiple tissues of the synovial joint and is characterised by articular cartilage degeneration and bone remodelling. Interferon-γ (IFN-γ) is implicated in osteoarthritis pathology exerting its biological effects via various mechanisms including activation of protein kinase R (PKR), which has been implicated in inflammation and arthritis. This study investigated whether treatment of articular cartilage chondrocytes and osteoblasts with IFN-γ could induce a degradative phenotype that was mediated through the PKR signalling pathway. IFN-γ treatment of chondrocytes increased transcription of key inflammatory mediators (TNF-α, IL-6), matrix degrading enzymes (MMP-13), the transcription factor STAT1, and PKR. Activation of PKR was involved in the regulation of TNF-α, IL-6, and STAT1. In osteoblasts, IFN-γ increased human and mouse STAT1, and human IL-6 through a mechanism involving PKR. ALP, COL1A1 (human and mouse), RUNX2 (mouse), and PHOSPHO1 (mouse) were decreased by IFN-γ. The number of PKR positive cells were increased in post-traumatic OA (PTOA). This study has revealed that IFN-γ propagates inflammatory and degenerative events in articular chondrocytes and osteoblasts via PKR activation. Since IFN-γ and PKR signalling are both activated in early PTOA, these mechanisms are likely to contribute to joint degeneration after injury and might offer attractive targets for therapeutic intervention.

## Highlights

•IFN-γ treatment of chondrocytes increased transcription of TNF-α, IL-6, and STAT1 via PKR activation.
•In osteoblasts, IFN-γ increased STAT1 and IL-6 via PKR activation.
•The number of PKR positive cells were increased in post-traumatic OA (PTOA).
•IFN-γ propagates inflammatory and degenerative events in articular chondrocytes and osteoblasts via PKR activation.
•IFN-γ and PKR signalling are both activated in early PTOA and are likely to contribute to joint degeneration after injury.

## 1 Introduction

Osteoarthritis (OA) is a heterogeneous disease affecting multiple tissues of the synovial joint and is characterised by articular cartilage degeneration, synovial inflammation and bone remodelling, with chronic inflammation promoting disease symptoms and accelerating disease progression [1]. Cytokines such as tumour necrosis factor-alpha (TNF-α), interleukin-1 (IL-1), IL-6 and interferon-gamma (IFN-γ) and proteolytic enzymes such as a disintegrin and metalloproteinase with thrombospondin motifs −4 and −5 (ADAMTS-4 and -5) and matrix metalloproteinases (MMPs) are produced by joint tissues and immune cells and drive pathogenesis by promoting extracellular matrix catabolism to cause structural damage [[2], [3], [4], [5]].

IFN-γ is implicated in joint pathology; concentrations are increased in synovial fluids from patients with end-stage OA [6,7], focal cartilage defects [8], post-traumatic OA (PTOA) [9] and acute anterior cruciate ligament (ACL) injury [10,11]. IFN-γ exhibits both anti- and pro-inflammatory effects in joint tissues. It protects articular cartilage in models of rheumatoid arthritis [12] and inhibits IL-1 induced increases in MMP-13 in healthy but not OA chondrocytes [13,14]; it also increases nitric oxide, IL-6, reactive oxygen species and prostaglandin E2 (PGE2) release and downregulates collagen and proteoglycans synthesis by chondrocytes [[15], [16], [17]]. IFN-γ also acts on bone cells to modulate bone turnover, inhibit bone resorption [15] and osteoclast differentiation [18], and accelerates early differentiation of mesenchymal stem cells or primary osteoblasts to osteoblasts by increasing expression of runt-related transcription factor (Runx2) [19], whilst inhibiting osteoblast proliferation and calcification [20].

IFN-γ exerts its biological effects by binding to cell surface receptors and recruiting the transcription factor, Signal transducer and activator of transcription 1 (STAT1) to the nucleus, to regulate transcription ([21]; reviewed in Ref. [22]). IFN-γ also activates the mitogen-activated protein kinase (MAPK), phosphoinositide 3-kinase, and nuclear factor kappa beta (NFkB) signalling pathways [23] and is a potent activator of protein kinase R (PKR) [[24], [25], [26]]. Increased expression of PKR correlates with activation of IFN-STAT1 signalling [27]. PKR is constitutively expressed at low levels in many cell types where it is activated by stress signals, dsRNA, viral infection and pro-inflammatory cytokines [[28], [29], [30]] and acts as a major regulator of transcription, translation, cell growth, differentiation, metabolism, and apoptosis (reviewed in Ref. [31]), [27,32,33]. PKR dysregulation has been implicated in many inflammatory processes and age-related diseases (reviewed in Ref. [28]) [29], including arthritis, where PKR activation increases production of IL-6 and TNF-α [24,25], and promotes cartilage breakdown and bone remodelling [[34], [35], [36], [37], [38], [39], [40]]. PKR is likely to play an important role in both normal and diseased joints. Chondrocytes and osteoblasts, constitutively express high levels of PKR, which is further activated by pro-inflammatory cytokines [39,41,42]. Activation of PKR in chondrocytes *in vitro* increases the production of matrix degrading enzymes [[40], [41], [42]], and proteoglycan degradation [39,41]. Inhibition of PKR signalling in MC3T3-E1 osteoblast-like cells increased growth, reduced expression of type I collagen, osteopontin, and osterix, reduced alkaline phosphatase (ALP) activity and mineralisation [43], and increased IL-6, MMP-8 and MMP-13 expression [44]. Over activation of PKR, by *in vivo* deletion of its endogenous inhibitor p58^IPK^, results in joint degeneration, with extensive cartilage and sub-chondral bone loss, accompanied by heterotopic bone formation in the joint capsule [37]. PKR is also up-regulated in the synovium of rats induced with inflammatory arthritis; interestingly, inhibition of active PKR reduces paw swelling in this model [38]. In addition, cartilage and bone taken from human patients undergoing total knee replacement for end-stage OA show high levels of active PKR [37,40].

To our knowledge, no studies have investigated the role of PKR in IFN-γ signalling in bone or cartilage cells. Since PKR is a major mediator of inflammatory responses, is activated by IFN-γ and implicated in the onset and progression of arthritis, we hypothesised that the degenerative effects of IFN-γ are mediated via PKR signalling in osteoblasts and chondrocytes and that inflammation in early PTOA is associated with PKR activation. This study assessed the effect of inhibiting the PKR pathway on markers of articular chondrocyte and osteoblast homeostasis and inflammatory mediators, under normal conditions and following induction of inflammation by IFN-γ. To further elucidate the role of PKR in inflammatory joint degeneration, we assessed activation of the PKR pathway in PTOA since this has a well-defined inflammatory phase characterised by upregulation of pro-inflammatory cytokines such as IL-6, IL-17A and IFN-γ [9,45].

## 2 Materials & methods

### 2.1 Materials

Chemicals were from Merck (Sigma; Poole, UK) and Thermo Fisher Scientific (Invitrogen, Paisley, UK) unless otherwise stated and were of analytical grade or above. Recombinant human and mouse IFN-γ were purchased from Peprotech EC (London, UK) and recombinant bovine IFN-γ from R&D Systems (Abingdon, UK); cytokines were dissolved in phosphate buffered saline (PBS: pH 7.4) containing 0.1% bovine serum albumin (BSA). Chondrocyte culture medium consisted of Dulbecco's Modified Eagle's Medium (1:1 mixture of DMEM-Glutamax-I™ and Ham's F12 media) containing 10 mM HEPES pH 7.4, 100 U/mL penicillin, 100 μg/ml streptomycin, 50 μg/ml ascorbate-2-phosphate and supplemented with 1x Insulin-transferrin-sodium selenite (ITS) to maintain chondrocyte phenotype and prevent serum withdrawal activation of signalling pathways [35,46]. MC3T3-E1 culture media consisted of α-Minimum Essential Medium (MEM) containing GlutaMAX™-I, 100 U/mL penicillin, 100 μg/ml streptomycin, and 10% (V/V) foetal bovine serum (FBS). For mineralisation assays, media was supplemented with 50 μg/ml ascorbate-2-phosphate, 10^−8^ M Dexamethasone and 10 mM b-glycerophosphate [47]. To inhibit the activation of PKR, a potent selective small molecule inhibitor (Insolution™ PKRi; 1 μM in 0.002% DMSO) which binds to the ATP-binding site [39,48] was added to cell cultures.

### 2.2 Primary chondrocyte culture

Full depth articular cartilage was taken from the metacarpophalangeal joints (from ≥4 legs) of 7-day-old bovine calves within 6 h of slaughter using a scalpel. Chondrocytes were isolated by enzymatic digestion (0.1% (w/v) pronase; Roche Applied Science, Burgess Hill, UK) in media containing 5% FBS for 30 min followed by overnight incubation in 0.04% (w/v) type II collagenase in media containing 5% FBS at 37 °C in a humidified atmosphere of 5% CO2, 95% air. Following isolation, chondrocytes were seeded at 1.0x10^6^ cells per well of a 24-well plate and cultured for 48 h at 37 °C in 1.0 ml serum-free, ITS supplemented media.

#### 2.2.1 Biochemical treatments

After 48 h, the media was replaced, and chondrocytes cultured for 24 h prior to the direct addition of treatments to the existing media to prevent activation of the MAPK signalling pathway by changing the media [49]. Chondrocytes were stimulated ±10 ng/ml IFN-γ [12,50,51] for 48 h and either treated with PKRi (1 μM) or vehicle (DMSO 0.002% v/v) 30 min prior to the addition of IFN-γ (n=9/treatment group). At the end of the experiment, media were replaced with 1.0 ml of TRIzol and samples stored at −80°C.

### 2.3 Osteoblast culture

#### 2.3.1 MC3T3-E1 culture

Murine MC3T3 subclone 14 cells were used as an osteoblast-like cell. This cell line was chosen as it is well characterised as a mineralising clone of the MC3T3-E1 cell line displaying a stable osteoblastic phenotype [52]. Cells were cultured between passages 15–17, seeded at 3 x 10^4^ cells/ml in 24-well plates and cultured for 24 h at 37 °C in 1 ml media.

#### 2.3.2 Primary human osteoblast culture

In addition to analysing a stable osteoblast cell line we assessed the effect of IFN-γ on primary human osteoblast (HOBs) cultures. HOBS were prepared from waste bone fragments taken from a patient undergoing total knee replacement for osteoarthritis following informed consent (Ethics no.1O/MRE09/28) [53]. Briefly, bone fragments were placed in MEM containing 10% FBS and incubated at 37°C to allow osteoblasts to migrate out. Media was replaced twice weekly, and cells removed by trypsin digestion prior to expansion. Cells at passage 2 were seeded at 3 x 10^4^ cells/ml in 24-well plates and cultured for 24 h at 37 °C in 1 ml media.

#### 2.3.3 Biochemical treatments

After 24 h, the media was replaced with media containing either PKRi (1 μM) or vehicle (DMSO 0.002% v/v) and cells cultured for 30 min prior to the addition of IFN-γ (0 or 10 ng/ml; [50]) for 5-days (RNA analysis; n=3–6 wells/treatment) or 21-days (mineralisation assay; n=4–6 wells/treatment). Media and treatments (IFN-γ, PKRi, vehicle) were replenished every 3-days. At the end of the experiment, media were either replaced with 1.0 ml of TRIzol and samples stored at −80°C or wells assayed for mineralisation.

### 2.4 Induction of post-traumatic osteoarthritis (PTOA)

Procedures were performed in accordance with the U.K. Animals (Scientific Procedures) Act 1986 [Home Office licences 30/2959 and P287E87DF] and Directive 2010/63/EU of the European Parliament [45,54]. Briefly, 4 male mice were anaesthetised and a single 12 N load applied to the right hind limb resulting in ACL rupture; contralateral knees served as controls [45]. Female mice were excluded from the current study because of known female hormone effects on cartilage and bone physiology. Mice were culled after 21-days. Hind limbs were immediately fixed post-mortem in formalin at an orientation of 90° (2 days, 10% neutral buffered formalin), decalcified for 2-weeks (4°C, 10% EDTA), and embedded frontally in paraffin blocks for coronal sectioning parallel to the tibia. Serial sections (5 μm) obtained from approximately the centre of the joint, were dewaxed and rehydrated prior to staining with Toluidine Blue or processing for immunohistochemistry.

### 2.5 Quantitative RT-PCR analysis of gene expression

Total RNA was extracted using TRIzol from chondrocytes, MC3T3-E1 cells and human primary osteoblasts according to the manufacturer's instructions with the following exceptions. After the addition of chloroform, the entire RNA extraction mix was transferred to a tube containing Heavy Phase-lock Gel™ (Eppendorf®; VWR International) and centrifuged (13,000 g for 2 min at 4 °C). The upper aqueous layer was removed to a new Eppendorf tube, an equal volume of isopropanol added, and the RNA left to precipitate overnight at −20 °C. At the end of the extraction protocol, the RNA was DNase treated to remove genomic DNA (Ambion; Applied Biosystems, Warrington, UK) and re-suspended in 50 μL RNase-free water. RNA integrity and concentration were assessed by Nanodrop™. cDNA was generated in a 20 μL reaction from 500 ng RNA using 250 ng random hexamers (Promega) and Superscript III reverse transcriptase (200 units; Invitrogen). Gene expression was measured by SYBR green quantitative RT-PCR (RTqPCR) using the MX3000P™ qPCR system according to manufacturer's instructions (Stratagene®; Agilent Technologies UK, Stockport, UK) with 200 nM forward and reverse primers (Suppl. Table 1) annealing at 60 °C, unless otherwise stated. Genes represented markers for osteogenesis (Runx2, Col1A1, Phospho1, ALP, Smpd3, OCN), catabolism (MMP13), inflammation (TNFα, IL6) and signalling (PKR, Stat1). Reference genes, PP1A, YWHAZ, RPL4, 18S, b-actin, GAPDH were tested for stability across experimental conditions. The geometric mean of PP1A and YWHAZ for chondrocyte analysis (stability value 0.85), 18S and b-actin for MC3T3 analysis (stability value 1.017) and YWHAZ and 18S for human osteoblast analysis (stability value 0.894) were identified by RefFinder [55] as the most stable and used to calculate fold changes relative to untreated cells using the ΔΔCT method [56,57]. Primers were validated using a standard curve of five serial cDNA dilutions with primer efficiencies between 90 and 110% [58].

**Table 1:** Summary of IFN-induced effects.

(A) Chondrocytes		
	Regulated by IFN-γ	Effect of PKR inhibition
*Pkr* mRNA	↑	None
*Stat1* mRNA	↑	↓
*Tnfa* mRNA	↑	↓ to baseline
*Il6* mRNA	↑	↑↑ above baseline
*Mmp1*3 mRNA	↑	None
*Adamts4* mRNA	No	None
(B) MC3T3-E1 osteoblast-like cells		
	Regulated by IFN-γ	Effect of PKR inhibition
*Pkr* mRNA	No	↑ above baseline
*Stat1* mRNA	↑↑	None
*Il6* mRNA	No	None
*Runx2* mRNA	↓	None
*Ocn* mRNA	No	↑ above baseline
*Smpd3* mRNA	No	↓ below baseline
*Phospho1* mRNA	↓	↑↑ above baseline
*Alp* mRNA	↓	↑↑ above baseline
*Col1a1* mRNA	↓↓	↑ to baseline
Mineralisation	↑	↑↑
(C) Human primary osteoblasts		
	Regulated by IFN-γ	Effect of PKR inhibition
*PKR* mRNA	No	None
*STAT1* mRNA	↑	↓
*IL6* mRNA	↑↑	↓
*RUNX2* mRNA	No	↓ below baseline
*OCN* mRNA	↓	None
*SMPD3* mRNA	↑	↑↑
*ALP* mRNA	↓	None
*COL1A* mRNA	↓	None
Mineralisation	↓	↑↑

### 2.6 Mineralisation assay

For mineralisation assays, cells (MC3T3-E1 or HOBs) were cultured as described previously and media supplemented after 24hrs with 50 μg/ml ascorbate-2-phosphate, 10^−8^M Dexamethasone and 10 mM b-glycerophosphate for 21 days. At the end of 21-days, media was removed, and cells washed with PBS before being fixed in neutral buffered formalin for 15 min. Cells were washed with dH_2_O and stained with Alizarin Red S for 5 min. Stain was removed, mineralised cells photographed using a Samsung Galaxy S9 mobile phone, and cells washed 5x with 50% (v/v) ethanol before air drying. Colour was removed overnight from each well with 10% (w/v) cetylpyridnium chloride and absorbance read at 540 nm using a BMG-Labtech plate reader.

### 2.7 Immunocytochemistry

Immediately after collagenase isolation as in 2.2, chondrocytes were seeded onto 8 well glass chamber slides (0.5 x 10^6^ cells/well) and left for 48 h prior to stimulation as described in 2.2.1. Following treatment, cells were fixed in 2% (w/v) paraformaldehyde and permeabilised in 0.2% (v/v) Triton-X100. Each subsequent step was performed at room temperature unless stated otherwise and between each incubation step, sections were washed 3x 5 min in 0.01 M PBS containing 0.001% tween 20 (wash buffer). All antibodies were diluted in wash buffer. Cells were washed before blocking in 2% (v/v) normal goat serum (Dako, UK Ltd, Ely, UK) for 1 h. After overnight incubation at 4°C with a rabbit monoclonal phospho-specific primary antibody to active PKR [pT^446^] (1:100; Abcam [59]), cells were washed before incubating for 1 h with FITC-conjugated anti-rabbit antibody (9 μg/ml; Molecular probes, Invitrogen). Cells were washed and blocked as before prior to incubation with a monoclonal antibody to Golgi matrix protein (0.5 μg/ml anti-GM130; BD Transduction Laboratories™). Cells were washed and a goat anti-mouse Alexa 594 conjugated secondary antibody (5 μg/ml; Molecular probes, Invitrogen) applied to the cells for 1 h. Finally, after washing, cells were mounted in VECTASHIELD® Mounting Medium containing DAPI (1.5 μg/ml) to counterstain DNA (Vector Laboratories Ltd, Peterborough, UK). Representative and randomly selected cells from multiple fields of view were scanned with a Leica TCS SP2 confocal microscope (Leica, Heidelburg) using a 63× oil immersion objective lens (x7.5 zoom) as described previously [60]. To eliminate the possibility of spectral bleed-through between fluorescent probes, representative regions were scanned using appropriate excitation and emission settings for *sequential* recordings of DAPI (ex max 358 nm; em max: 461 nm), FITC (ex max: 494 nm; em max: 518 nm) and Alexa 594 (ex max:594 nm; em max: 617 nm). Maximum intensity, 3D projections were obtained by processing stacks of optical sections through the full depth of chondrocytes at a spacing of 0.5 μm. Since PKR is constitutively activated in sub-populations of chondrocytes *in vitro*, untreated cultures served as positive controls. Negative controls where the primary antibody was omitted were devoid of fluorescent signal.

### 2.8 Immunohistochemistry

Active PKR was immunolocalised in sections from uninjured and ACL ruptured mouse knees (n=1 section from n=4 mice) using a rabbit polyclonal antibody to phosphorylated PKR (Santa Cruz: sc-101783 [pT^446^]; 2 μg/ml). Sections were deparaffinised and rehydrated prior to antigen retrieval (1 mg/ml trypsin for 1 h at 37°C). Each subsequent step was performed at room temperature unless stated otherwise and between each incubation step, sections were washed 3x 5min in 0.01 M PBS, pH 7.4 containing 0.001% (v/v) Tween 20 (wash buffer). All antibodies were diluted in wash buffer. Endogenous peroxidase activity was blocked with 0.3% (v/v) hydrogen peroxide for 30 min. Sections were subsequently treated with 10% normal goat serum for 1 h prior to overnight incubation (4°C) with primary antibody, rabbit IgGs or PBS. Biotinylated secondary antibody was applied and incubated for 30 min before detection (Vectastain Elite ABC kit, nickel enhanced diaminobenzidine, Vector Laboratories). Sections were dehydrated, cleared in xylene and mounted before viewing on a Leica DMRB microscope. IgG and PBS controls were negative [45].

### 2.9 Data analysis

Graphs show individual data points, box and whisker plots of minimum and maximum values, 25th and 75th quartiles and median. Data were tested for normality and equal variances and transformed where necessary prior to ANOVA and appropriate *post hoc* tests as indicated (Minitab 18). Differences were considered significant at *p=0.05*. For all statistics, unless stated otherwise, treatments were compared to vehicle controls.

## 3 Results

### 3.1 The role of IFN-γ and PKR in chondrocytes

#### 3.1.1 Regulation of genes involved in chondrocyte signalling, inflammation, and catabolism

**Fig. 1:**
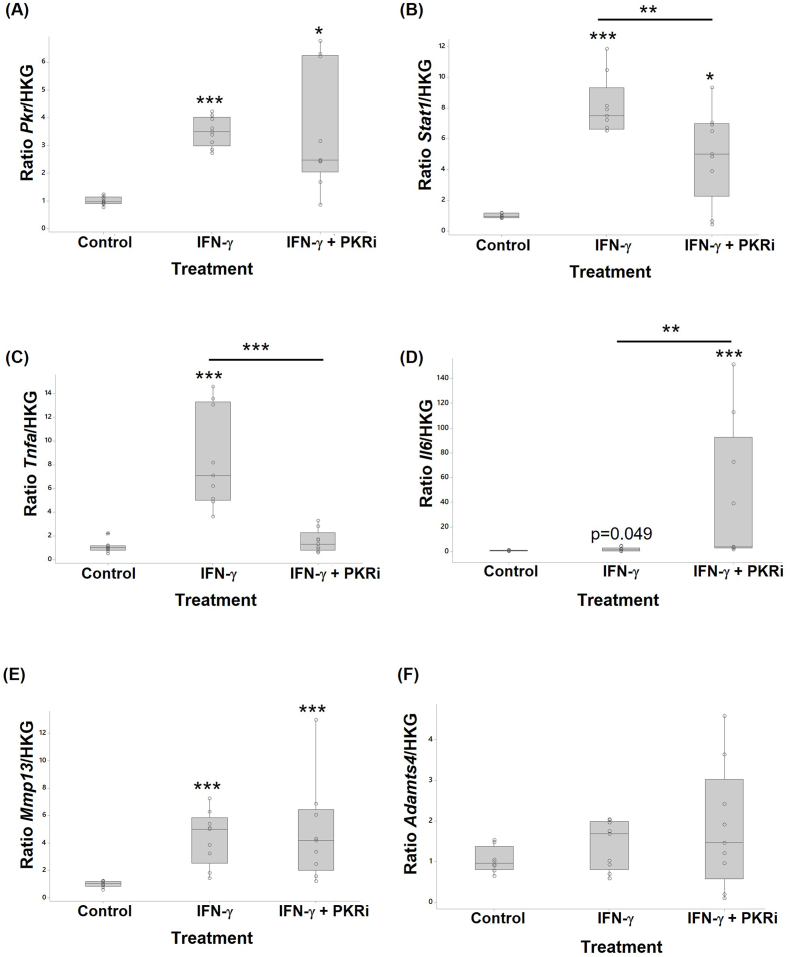
IFN-γ and PKRi regulate chondrocyte gene expression. Primary bovine chondrocytes treated with 10 ng/ml IFN-γ, or 10 ng/ml IFN-γ + 1.0 μM PKRi were compared with control cultures (vehicle, 0.002% DMSO). RNA from cells (n = 9 wells per treatment) were analysed by RTqPCR to determine the relative expression of (A) *Pkr*, (B) *Stat1*, (C) *Tnfa*, (D) *Il6*, (E) *Mmp13*, and (F) Adamts4 mRNA. Data are presented as fold change relative to control cells calculated using the ΔΔCT method. Significant differences were detected by one-way ANOVA and Fisher's post hoc test: *p ≤ 0.05; **p < 0.01; ***p < 0.001.

The effect of IFN-γ, in the presence or absence of PKR inhibition, on markers of chondrocyte signalling, catabolism, and inflammation was assessed by RTqPCR (Fig. 1). IFN-γ treatment increased *Pkr* (ranked data; 3.2-fold p<0.001), *Stat1* (ranked data; 8-fold p<0.001), *Tnfa* (log data; 7.7-fold p<0.001), *Il6* (ranked data; 2-fold p=0.049), and *Mmp13* (log data; 4.3-fold p<0.001) gene expression. IFN-γ treatment had no effect on *Adamts4* gene expression. PKRi co-treatment reduced the IFN-γ -induced increase in *Tnfa* (log data; 5.4-fold p<0.001) and *Stat1* (ranked data; 1.6-fold p=0.004) expression but had no effect on expression of *Pkr* or *Mmp13* and increased the expression of *Il6* (ranked data; 21-fold p=0.002).

Inhibition of PKR in the absence of IFN-γ treatment had no effect on chondrocyte basal *Pkr*, *Stat1*, *Il6*, or *Tnfa* gene expression but resulted in a 3.4-fold reduction in basal *Mmp13* levels (p=0.001) and a 1.6-fold reduction in *Adamts4* levels (p=0.007) (Suppl. Table 2A).

#### 3.1.2 Active PKR localises to the Golgi in chondrocytes stimulated with IFN-γ

**Fig. 2:**
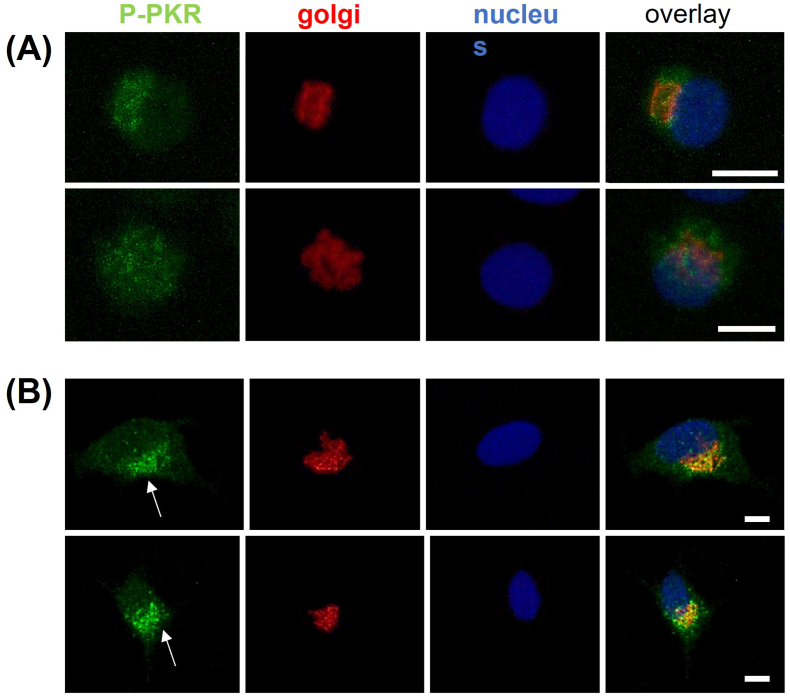
Active PKR localises to the Golgi in chondrocytes stimulated with IFN-γ The subcellular localisation of phosphorylated PKR was determined by immunocytochemistry. Representative cells chosen randomly from multiple fields of view were imaged by confocal microscopy with appropriate settings for FITC (green), Alexa 594 (red), and DAPI nuclear counterstain (blue). Maximum intensity 3D reconstructions are shown. Staining of phosphorylated PKR (green) was observed throughout the cytoplasm of untreated, control cells (A) and was concentrated into distinct pools that co-localised with the Golgi (red) following IFN-γ treatment (B). Scale bar = 5 μm. (For interpretation of the references to colour in this figure legend, the reader is referred to the Web version of this article.)

Chondrocytes were labelled for phosphorylated (active) PKR (Fig. 2). Control cells showed a rounded phenotype typical of normal chondrocytes. In unstimulated cells, diffuse staining for phosphorylated PKR was observed with some co-localisation with the Golgi marker, GM130 (Fig. 2A). In IFN-γ stimulated cultures, cells appeared less rounded and a more concentrated perinuclear pool of phosphorylated PKR co-localising with GM-130 was observed (arrows, Fig. 2B).

### 3.2 The role of IFN-γ and PKR in osteoblasts

#### 3.2.1 Regulation of genes involved in signalling and inflammation

**Fig. 3:**
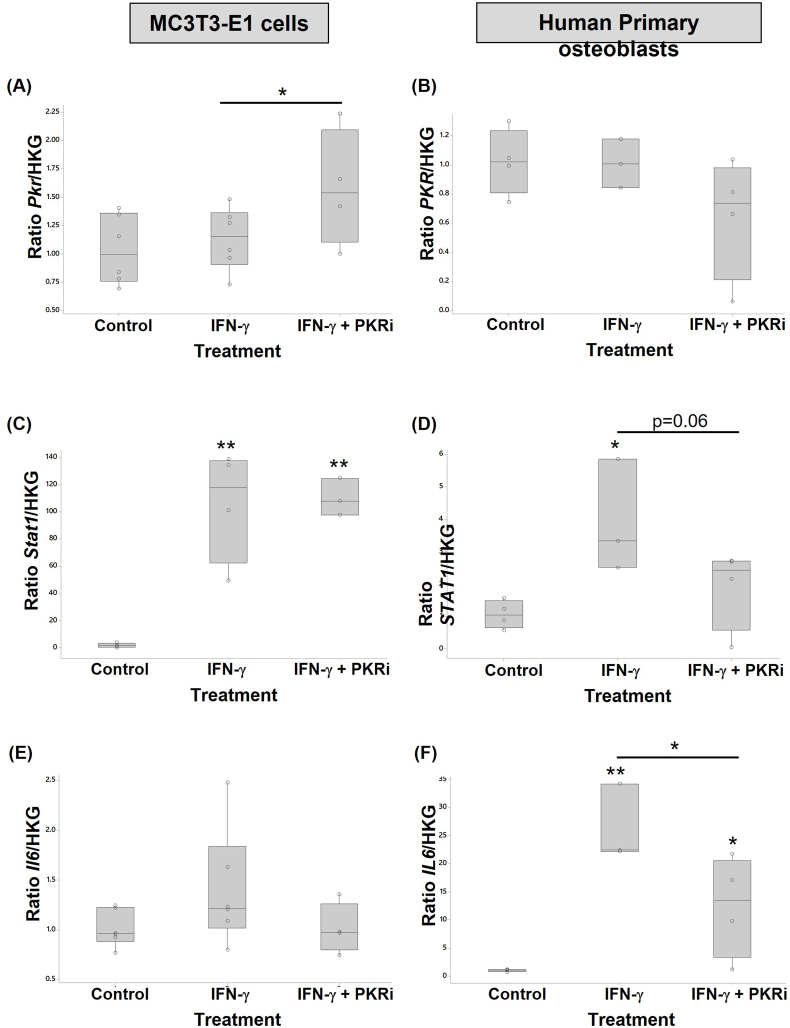
IFN-γ and PKRi regulate osteoblast gene expression. Murine MC3T3-E1 cells (A, C, E) and human primary osteoblasts (B, D, F) treated with 10 ng/ml IFN-γ, or 10 ng/ml IFN-γ + 1.0 μM PKRi were compared with control cultures (vehicle, 0.002% DMSO) (n = 3–6 per treatment). RNA extracted from cells was analysed by RTqPCR to determine the relative expression of (A & B) IL6, (C & D) PKR, (E & F) STAT1 mRNA. Data are presented as fold change relative to control cells calculated using the ΔΔCT method. Significant differences were detected by one-way ANOVA and Fisher's post hoc test: *p ≤ 0.05; **p < 0.01.

The effect of IFN-γ treatment in the presence or absence of PKR inhibition on markers of cell signalling and inflammation was assessed by RTqPCR (Fig. 3). IFN-γ treatment did not affect *Pkr* or *Il6* gene expression in MC3T3-E1 cells but significantly increased *Stat1* gene expression (ranked data; 57-fold p=0.004; this was not returned to baseline by co-treatment with PKRi. IFN-γ treatment had no effect on *PKR* levels in human osteoblasts but significantly increased *STAT1* (3.6-fold; p=0.015) and *IL6* (25.8-fold; p=0.001) expression. The addition of PKRi reduced mean *STAT1* (2-fold; p=0.06) and *IL6* (2-fold; p=0.023) levels although not to baseline.

PKRi treatment alone reduced basal *Il6* levels (1.7-fold p=0.003) expression in MC3T3-E1 cells (Suppl. Table 2B). No affect was observed on basal *STAT1* or *PKR* expression (data not shown). In HOBS, PKRi treatment alone reduced basal *PKR* (2.9-fold p=0.005), and *STAT1* (2.64-fold p=0.054) gene expression (Suppl. Table 2C). No affect was observed on basal *IL6* expression (data not shown).

#### 3.2.2 Regulation of genes involved in osteogenesis

**Fig. 4:**
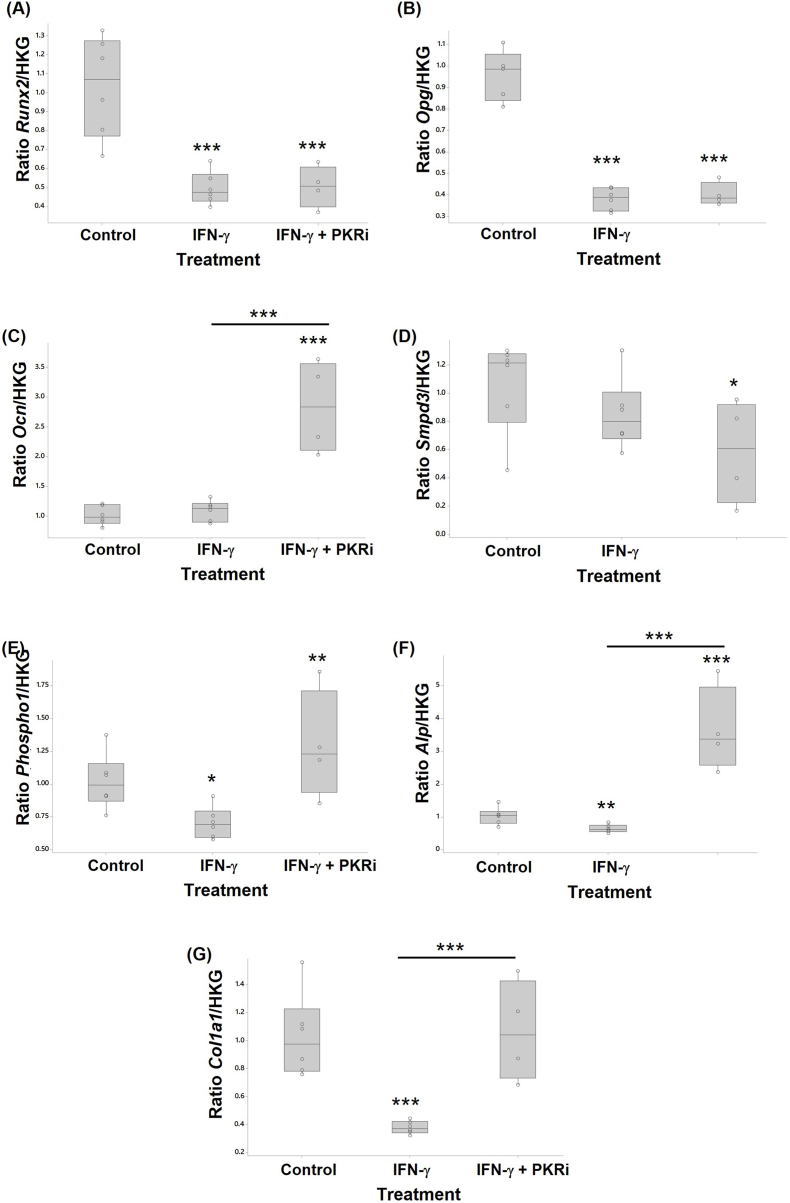
IFN-γ and PKRi regulate genes involved in murine MC3T3-E1 osteogenesis. Murine MC3T3-E1 cells treated with 10 ng/ml IFN-γ, or 10 ng/ml IFN-γ + 1.0 μM PKRi were compared with control cultures (vehicle, 0.002% DMSO) (n = 3–6 per treatment). RNA extracted from cells was analysed by RTqPCR to determine the relative expression of (A) *Runx2*, (B) *Ocn*, (C) *Smpd3*, (D) *Phospho1*, (E) *Alp*, and (F) *Col1a1* mRNA. Data are presented as fold change relative to control cells calculated using the ΔΔCT method. Significant differences were detected by one-way ANOVA and Fisher's post hoc test: *p ≤ 0.05; **p < 0.01; ***p < 0.001.

**Fig. 5:**
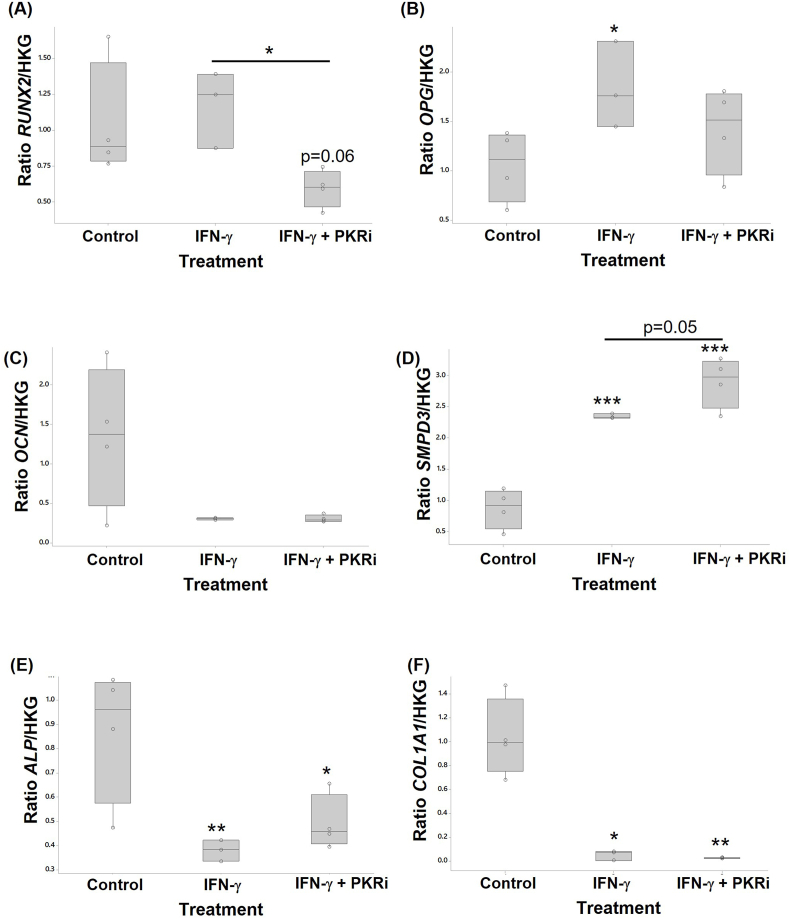
IFN-γ and PKRi regulate genes involved in human primary osteoblast osteogenesis. Primary human osteoblasts treated with 10 ng/ml IFN-γ, or 10 ng/ml IFN-γ + 1.0 μM PKRi were compared with control cultures (vehicle, 0.002% DMSO) (n = 3–6 per treatment). RNA extracted from cells was analysed by RTqPCR to determine the relative expression of (A) *RUNX2*, (B) *OCN*, (C) *SMPD3*, and (D) *COL1A1* mRNA. Data are presented as fold change relative to control cells calculated using the ΔΔCT method. Significant differences were detected by one-way ANOVA and Fisher's post hoc test: *p ≤ 0.05; **p < 0.01; ***p < 0.001.

The effect of IFN-γ treatment in the presence or absence of PKR inhibition on markers of osteogenesis was assessed by RTqPCR from RNA extracted from MC3T3E-1 cells (Fig. 4) and human osteoblasts (Fig. 5).

##### 3.2.2.1 MC3T3-E1 cells

Treatment of MC3T3-E1 cells with IFN-γ decreased the expression of *Runx2* (log data 2-fold p<0.001), *Opg* (2.5-fold p<0.001), *Phospho1* (1.45-fold p=0.049), *Alp* (log data 1.6-fold p=0.008), and *Col1a1* (log data 2.7-fold p<0.001). IFN-γ treatment did not affect *Ocn* or *Smpd3* expression. The addition of PKRi had no effect on the IFN-induced reduction in *Runx2* or *Opg* levels. However, IFN-γ and PKRi co-treatment increased *Ocn* (2.8-fold p<0.001 vs control), *Alp* (3.5-fold p<0.001 vs control) and *Phospho1* (1.3-fold p=0.003 vs control) expression, restored *Col1a1* levels to baseline but reduced *Smpd3* levels (1.8-fold p=0.034).

PKRi treatment alone reduced *Runx2* (2-fold p=0.008), *Opg* (1.86-fold p<0.001), and *Smpd3* (1.6-fold p=0.05) expression but increased *Ocn* (2.2-fold p=0.008), *Phospho1* (2.2-fold p=0.031) and *Alp* (3.9-fold p=0.001) (Suppl. Table 2B).

##### 3.2.2.2 Human primary osteoblasts

IFN-γ treatment of HOBS had no effect on *RUNX2* expression but increased *OPG* (1.7-fold p=0.036) and *SMPD3* (2.7-fold p<0.001). In contrast, IFN-γ significantly decreased *ALP* (2.3-fold p=0.009) and *COL1A1* (ranked data 20.7-fold p=0.012) expression. The addition of PKRi to IFN-γ treated cells resulted in a reduction in *RUNX2* (2-fold p=0.03 vs IFN-γ alone) but did not alter *OCN*, *SMPD3*, *ALP*, and *COL1A1* levels compared to IFN-γ alone. *PHOSPHO1* mRNA was not detected in human osteoblasts.

The addition of PKRi alone only significantly affected basal *RUNX2* expression resulting in a 6-fold reduction (p=0.028); mean levels of *OPG* (1.82-fold p=0.079), and *COL1* (1.76-fold p=0.072) were reduced (Suppl. Table 2C). Basal *OCN*, *SMPD3*, and *ALP* levels were not affected by PKRi alone (data not shown).

#### 3.2.3 Inhibition of PKR regulates mineralisation

**Fig. 6:**
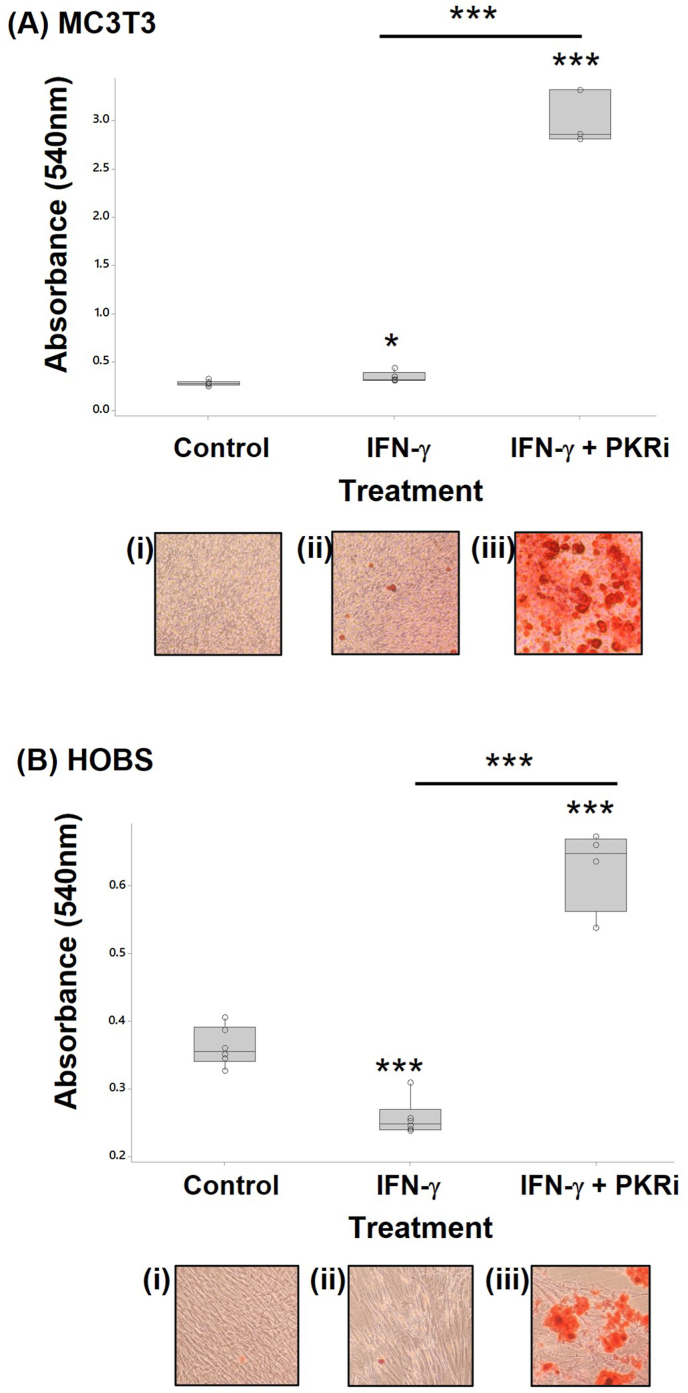
**IFN-γ and PKRi regulate *in vitro* mineralisation of murine MC3T3-E1 cells and human primary osteoblasts.** MC3T3-E1 (A) and human primary osteoblasts (B) were cultured under mineralising conditions; cells were treated with (i) 0.002% DMSO (ii) 10 ng/ml IFN-γ, or (iii)10 ng/ml IFN-γ + 1.0 μM PKRi; n = 4–6 per treatment. After 21-days media was removed and cells fixed and stained with Alizarin Red S for 5 min. Colour was removed and absorbance read at 540 nm using a plate reader. Significant differences were detected by one-way ANOVA and Fisher's post hoc test: *p ≤ 0.05; ***p < 0.001. (For interpretation of the references to colour in this figure legend, the reader is referred to the Web version of this article.)

Mineralisation in osteoblasts was assessed by alizarin red staining (Fig. 6). IFN-γ treatment of MC3T3-E1 cells increased mineralisation compared to control cells (log data; 1.2-fold p=0.012) but reduced mineralisation in human osteoblasts (log data; 1.4-fold p<0.001). Mineralisation was increased when PKRi was added to IFN-γ containing cultures of MC3T3-E1 (8.7-fold p<0.001) and in human osteoblasts (2.4-fold p<0.001). This increased mineralisation was due to PKRi since PKRi alone increased alizarin red staining in both MC3T3-E1 (log data; 10.96-fold p=0.002) and primary human osteoblast (12.4-fold p<0.001) cells (Suppl. fig. 1).

### 3.3 Active PKR is present in areas of bone remodelling in a mouse *in vivo* model of PTOA

**Fig. 7:**
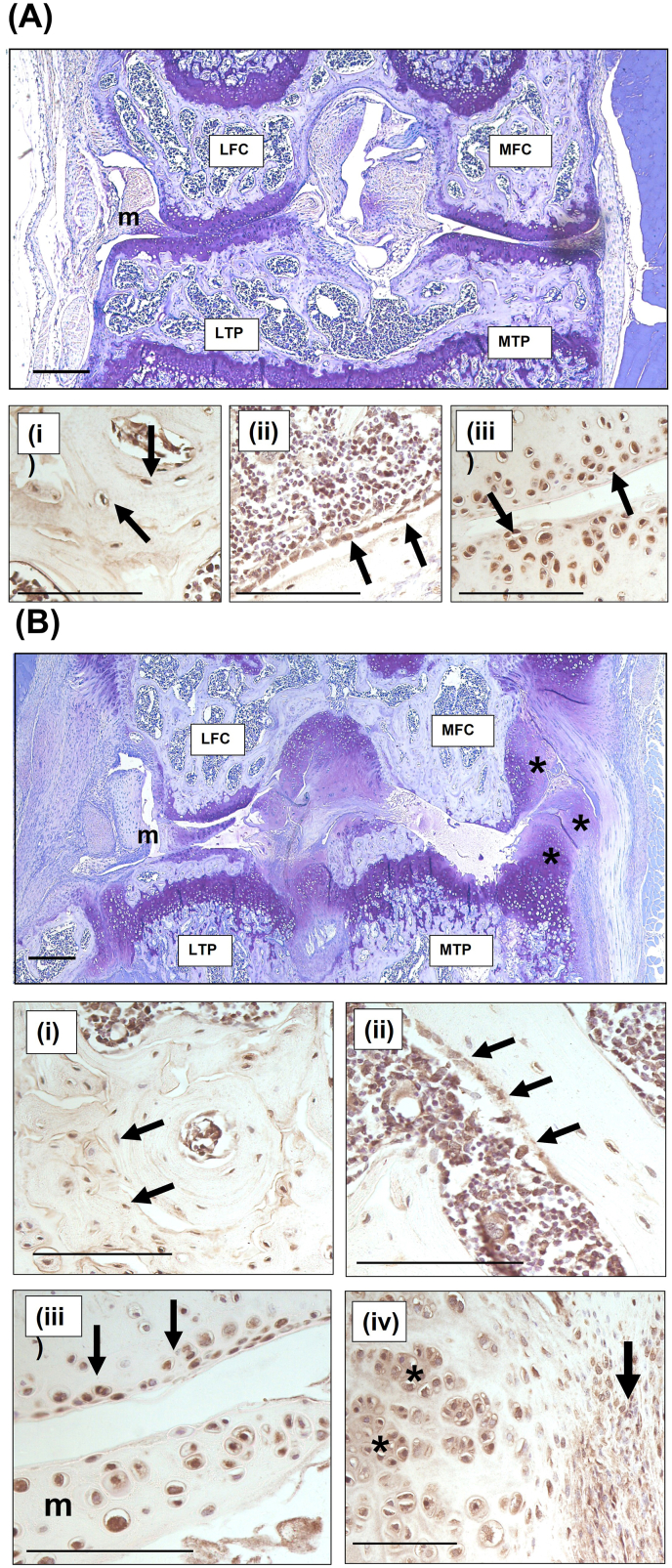
**Active PKR is prevalent in areas of inflammation and bone remodelling in PTOA.** Sections were taken from (A) uninjured and (B) injured knees of mice culled at 21‐days post‐ACL rupture. Toluidine blue stained consecutive sections from a contralateral control limb and injured limb show the extent of degeneration. Phosphorylated (active) PKR was localised by immunohistochemistry (i-iv) and found to be located throughout the joint in uninjured limbs (A), staining in osteocytes (i), osteoblasts and bone marrow cells (ii), and articular chondrocytes (iii). In injured limbs (B), osteocytes (i), osteoblasts and bone marrow cells (ii) and articular chondrocytes within the remaining lateral cartilage (iii) stained positive for active PKR. In addition, synovial infiltrate (iv; black arrows) and developing osteophytes (iv; asterix) stained positive for active PKR. LTP, lateral tibial plateau, LFC, lateral femoral condyle; MTP, medial tibial plateau; MFC, medial femoral condyle; m, meniscus. Scale bar = 500 μM (A & B) and 50 μM (i-iv). (For interpretation of the references to colour in this figure legend, the reader is referred to the Web version of this article.)

Phosphorylated PKR immunolocalised to a large number of cells in both uninjured and injured knees of mice culled at 21‐days post‐ACL rupture was determined by immunohistochemistry (Fig. 7). Active PKR was located throughout control limbs (Fig. 7A) with staining in osteocytes (panel i), osteoblasts and bone marrow cells (panel ii), and articular chondrocytes (panel iii). Likewise, after ACL rupture (Fig. 7B), active PKR was also located within the osteocytes (panel i) and osteoblasts and bone marrow cells (panel ii), remaining chondrocytes on the lateral femoral condyle and tibial plateau (panel iii) as well as in inflammatory cells within the synovium and cells within the developing osteophytes that arose after ACL rupture (panel iv). Extensive inflammation, cartilage loss and bone remodelling were only observed only in injured knees (Fig. 7B).

## 4 Discussion

IFN-γ plays a fundamental role in bone and cartilage homeostasis mediating immune and inflammatory responses [23], modulating the metabolism of connective tissue cells [15,17] and being implicated in the pathogenesis of joint disease and following joint injury [[6], [7], [8], [9], [10], [11],[61], [62], [63]]. PKR signalling is activated in early OA and propagates pro-inflammatory cytokine signals [[34], [35], [36],^39^,^40^] however, little is known about the role of PKR in IFN-induced osteoblast and chondrocyte signalling. We therefore investigated the effect of IFN-γ on articular chondrocytes and osteoblasts, key mediators of cartilage and bone function and determined whether these cellular responses were mediated through PKR; these findings are summarised in Table 1.

### 4.1 The effect of IFN-γ on chondrocytes

IFN-γ treatment of chondrocytes increased transcription of key inflammatory mediators (TNF-α, IL-6), matrix degrading enzymes (MMP-13), the transcription factor STAT1, and PKR. In response to IFN-γ, STAT1 forms homodimers which translocate to the nucleus and bind to GAS elements in the promoters of target genes [23]. Several studies have shown cytokine activation of STAT1 in chondrocytes [13,[64], [65], [66], [67]]. *TNFa* was strongly upregulated by IFN-γ treatment of chondrocytes consistent with its rapid induction by IFN-γ during an inflammatory response in other cell types [24,25]. IFN-γ treatment of chondrocytes also increased *IL-6* expression, but in this case only 2-fold. The effect of IFN-γ on *IL6* gene transcription has previously been shown to be cell type dependant; in monocytes [68] and RPE cells additional inducers such as TNFα and LPS are required for IFN-γ upregulation of *IL6* [69] whereas in HeLa S3 cells, IFN-γ on its own was shown to strongly induce transcription of *IL6* [70]. IL-6 correlates with disease activity and joint destruction in rheumatoid arthritis (reviewed in Ref. [71], is upregulated during the inflammatory phase of OA [3] and implicated in the pathogenesis of PTOA [45]. MMP-13, one of the main type II collagen cleaving enzymes, is considered a major effector enzyme in OA since increases in its activity will affect the irreversible loss of ECM architecture and function [72,73]. Our finding that IFN-γ treatments increases MMP-13 expression in chondrocytes differs to those of Ahmad et al. (2007) who showed that IFN-γ treatment activated STAT1 in normal human chondrocytes to inhibit MMP-13 expression [13] although in OA chondrocytes, the effect on MMP-13 was reduced due to diminished levels of the IFN-γ R1 and thus impaired STAT1 signalling [14]. Our data is derived from chondrocytes taken from 7-day old, healthy calves, cultured serum-free but supplemented with ITS, treated with IFN-γ whereas Ahmad (2007) used human primary chondrocytes from an undefined age group, cultured in serum free conditions, treated with 300 U/ml IFN-γ. The discrepancy on the effect of IFN-γ on MMP-13 may therefore be due to disease status, species, or age of cells. In addition, the concentration of IFN-γ maybe different between studies since there is no protein reference standard available for the bovine IFN-γ used in this study to allow conversion to international units. IFN-γ has been shown to inhibit IL1-induced increases in MMP-13 in synovial fibroblasts [12]. In contrast, IFN-γ treatment of chondrocytes inhibits nitric oxide, IL-6, IL-1ra, and PGE2 production, inhibits IL-8 and proteoglycan production [15,74], and impairs collagen synthesis [75]. Therefore, it is likely that IFN-γ has both anti- and pro-inflammatory/degenerative effects depending on the circumstances and cellular environment.

#### 4.1.1 The role of PKR

This study revealed an important role for PKR in regulating the expression of several key molecules known to be involved in the propagation of inflammation and OA pathogenesis. Corroborating data obtained from human hematopoietic progenitor cells [76] and mouse embryo fibroblasts [77], IFN-γ increased activation of PKR in primary bovine chondrocytes possibly by direct binding of the pseudoknot located within the mRNA structure of IFN-γ [78]. Notably, the IFN-γ induced increase in *TNFa* mRNA was completely blocked following treatment with the PKR inhibitor. TNF-α is expressed rapidly during an inflammatory response and is known to be induced by IFN-γ via activation of PKR resulting in the increased expression of inflammatory mediators [24,25]. The transcriptional events responsible for this increase are not known but may occur via IFN-induced upregulation of the master transcription factor, Interferon Regulatory Factor 1 (IRF-1) which binds to *TNFa* [79]. PKR also contains an IRF binding element in its gene so its expression could be directly, or in synergism with TNF, increased by IFNg-STAT1-IRF1 [80]. In addition, an element within *TNFa* pre-mRNA has been shown to act as a pseudoknot capable of binding to and activating PKR enhancing *TNFa* [35,81,82]; it seems likely that a positive feedback loop exists where IFN-γ activates TNFα via PKR, and TNF activates PKR, ultimately resulting in the induction of inflammation.

Interestingly, inhibition of PKR activity in the presence of IFN-γ in chondrocytes resulted in a large increase in *Il6* expression suggesting that active PKR suppressed the action of IFN-γ and prevented IFN-induced increases in IL-6. The actual mechanism of IFN-induced upregulation of IL-6 in chondrocytes however is not known and requires further study. The IFN-γ induced increase in Stat1 was, in part, mediated through PKR and is in keeping with previous studies showing that increased expression of PKR correlates with activation of IFN-STAT1 signalling [27].

Active PKR is clearly important in the regulation of cartilage turnover in normal joints since basal levels of active PKR are constitutively high in secretory cells such as chondrocytes and its inhibition resulted in a significant decrease in *Mmp13* and *Adamts4* genes. However, the IFN-γ induced increase in MMP-13 expression that we observed was not dependent on the PKR pathway.

### 4.2 The effect of IFN-γ on osteoblasts

IFN-γ treatment increased STAT1 expression but did not affect PKR transcription in human primary and mouse osteoblasts. Transcription of IL-6 was also increased but only in human osteoblasts. As discussed, IL-6 contributes to the pathogenesis of rheumatoid arthritis (reviewed in Ref. [71], OA [3] and PTOA (reviewed in Refs. [45,83]) and acts as a negative regulator of osteoblast differentiation [84]. The effects of IFN-γ on *Stat1* are likely to influence osteoblast differentiation. STAT1 has been shown to act as an inhibitor of osteoblast differentiation and is required for the IFN-γ mediated inhibition of bone growth; in the absence of STAT1, osteoblast differentiation and alkaline phosphatase (ALP) activity is enhanced [85]. In the current study, IFN-γ treatment significantly decreased Alp and Col1a1 gene expression in human and mouse osteoblasts supporting a role for IFN-γ as an inhibitor of osteoblast differentiation. Zha et al. described IFN-γ as a master regulator of differentiation showing that it reduced ALP, which is secreted during the early phase of differentiation, reduced Runx2, the master transcription factor for osteoblast differentiation, and reduced COL1A1 expression in dental pulp stem cells [67]. The MC3T3 osteoblasts showed a large reduction in Runx2 expression after IFN-γ treatment, although this was not the case for human primary osteoblasts. In contrast, others have reported that IFN-γ treatment increases MSC differentiation to osteoblasts, increases the expression of osteogenic genes such as *Runx2* and *Alp* and increases ALP activity and bone volume and that IFN-γ siRNA inhibits osteoblast differentiation [19,86,87]. In the current study, IFN-γ treatment significantly decreased Phospho1 gene expression and reduced mean Smpd3 gene expression in mouse osteoblasts supporting a role for IFN-γ in inhibition of osteoblast differentiation. Phospho1 functions with tissue-nonspecific alkaline phosphatase (TNAP) and neutral sphingomyelinase 2, encoded by the *Smpd3* gene to liberate inorganic phosphate for mineralisation and facilitate calcification [88,89], is expressed at high levels in subclone 14 of MC3T3-E1 cells and is required for osteoblast mineralisation [90]. Surprisingly, a small increase in alizarin red staining was observed following IFN treatment of MC3T3-E1 cells suggesting an increase in mineralisation in these cultures. This was unexpected and may represent ectopic mineralisation not associated with the matrix or dystrophic calcification given the IFN-γ induced reduction in osteogenic gene expression [91]. Unlike MC3T3-E1 cells, *PHOSPHO1* gene levels in human osteoblasts were below the limits of detection; the lower levels of PHOSPHO1 may explain why overall, levels of mineralisation were lower in human cells compared to the mouse cell line [90]. Interestingly, *SMPD3* gene levels were increased by IFN-γ treatment of human primary osteoblasts, which may reflect a compensatory rescue mechanism to ensure sufficient Pi generation in the face of reduced *PHOSPHO1* and *ALP*.

#### 4.2.1 The role of PKR

PKR was involved in the regulation of several IFN-γ induced molecules in osteoblasts dependent on cell source. IFN-γ did not regulate PKR expression in either mouse or human osteoblasts, and inhibition of PKR activation was not consistent in the 2 cell types. In human osteoblasts, the IFN-γ induced upregulation of IL-6 was PKR dependent implicating a role for PKR in this important inflammatory mediator. The mechanism of action may involve STAT1 and/or IRF-1 binding to their respective elements in the *IL6* gene [70], consistent with our data showing that the IFN-γ induced increase in *Stat1* expression in human osteoblasts was, in part, mediated through PKR. This contrasts with the studies of Yoshida et al. who showed that *Stat1* mRNA was increased in osteoblasts in the absence of active PKR [44]. The IFN-induced inhibition of mouse osteoblast differentiation was reversed by the addition of the PKR activation inhibitor which increased *Phospho1*, *Alp*, Ocn, and *Col1a1* gene expression and significantly increased mineralisation as detected by alizarin red staining. This was not the case in human osteoblasts where PKR inhibition, either in the presence or absence of IFN-γ, reduced *RUNX2* expression but had no effect on other osteogenic genes despite a marked increase in Alizarin red staining. The addition of PKRi to non-IFN treated MC3T3-E1 cells also resulted in an increase in the expression of *Phospho1*, *Alp*, and *Ocn* followed by a substantial increase in mineralisation (suppl. fig. 2) suggesting a negative role for PKR in osteoblast differentiation. This was different to the findings of Yoshida et al. who reported PKR to be a positive regulator of differentiation using dominant negative PKR mutants that lack the catalytic domain [43,44,92]. In the current study we used a PKR inhibitor that blocks the ATP binding site preventing activation which may produce different results to the dominant negative PKR mutants; additional studies are clearly required to assess these differences.

### 4.3 The role of PKR in PTOA

Finally, to assess whether active PKR may play a part in the pathogenesis of PTOA, which has a well-defined inflammatory phase characterised by upregulation of pro-inflammatory cytokines such as IL-6, IL-17A and IFN-γ [9,45] we looked for phosphorylated PKR in the knee joints from mice with PTOA. Active PKR was detected throughout the joint cells of both the injured and uninjured mice. Following induction of PTOA however, extensive inflammation within the synovium, large developing osteophytes and areas of bone remodelling were also found to have high levels of active PKR. Since activation of PKR in chondrocytes is known to increase levels of matrix degrading enzymes [[40], [41], [42]], and proteoglycan degradation [39,41] and over activation of PKR results in joint degeneration accompanied by heterotopic bone formation in the joint capsule [37] these findings implicate PKR signalling in the pathogenesis of PTOA.

### 4.4 Study limitations

The current study has several limitations. Although we found that IFN-γ increased phosphorylation of PKR in chondrocytes, we were unable to assess levels of active PKR in the osteoblast cells. In addition, although IFN-γ is reported to be increased in PTOA, levels of IFN-γ mRNA within our mouse model of PTOA are below the levels of detection and therefore cannot say whether the increased phosphorylated PKR observed in the mouse sections are due to enhanced IFN-γ signalling. We were unable to obtain chondrocytes and osteoblasts from a single species to assess the effects of IFN-γ. Several differences were observed between the mouse osteoblast cell line and human primary osteoblasts which may be due to differences in the species response to IFN-γ, differences in the cell line vs primary cell response or because the primary cells were taken from a patient with end-stage OA. Further studies are required to establish whether donor age, gender or disease status affects the primary HOB response to IFN-γ.

## 5 Conclusion

This study has revealed, for the first time, that IFN-γ propagates inflammatory and degenerative events in articular chondrocytes and osteoblasts via PKR activation. Since IFN-γ and PKR signalling are both activated in early PTOA, these mechanisms are likely to contribute to joint degeneration after injury and might offer attractive targets for therapeutic intervention.

## Abbreviations

ADAMTS a disintegrin and metalloproteinase with thrombospondin motifs, ALP alkaline phosphatase, ACL anterior cruciate ligament, BSA bovine serum albumin, DMEM Dulbecco's modified eagles medium, FBS foetal bovine serum, HOBs human osteoblasts, ITS insulin transferrin selenite, IFN interferon, IL Interleukin, MAPK mitogen-activated protein kinase, MMP matrix metalloproteinase, MEM Minimum Essential Medium, NFkB nuclear factor kappa beta, OA osteoarthritis, PGE2 prostaglandin E2, PBS phosphate buffered saline, PTOA post-traumatic osteoarthritis, PKR protein kinase R, RUNX2 runt-related transcription factor, STAT1 Signal transducer and activator of transcription 1, TNF-α tumour necrosis factor alpha.

## CRediT authorship contribution statement

**S.J. Gilbert:** Conceptualization, Methodology, Formal analysis, Investigation, Writing – original draft, Writing – review & editing, Visualization. **E.J. Blain:** Methodology, Writing – review & editing.

## Appendix A Supplementary data

Supplementary table 1 Quantitative qPCR primers. Supplementary table 1

Supplementary table 2 The effect of PKRi on basal gene expression was determined in (A) bovine chondrocytes (b) murine MC3T3-E1 cells, and (C) human primary osteoblasts. Cells were treated with 0.002% DMSO, or 1.0 μM PKRi (n = 3–6 per treatment) and RNA extracted from cells prior to analysis by quantitative PCR. Data are presented as fold change relative to control cells calculated using the ΔΔCT method (±S.E.M). Significant differences were detected by (A) Mann Whitney test or (B and C) Student's 2-sample test. Supplementary table 2

Supplementary figure 1 The effect of PKRi on basal mineralisation in (A) murine MC3T3-E1 and (B) human primary osteoblast cells. Cells were cultured under mineralising conditions for 21-days and treated with (i) 0.002% DMSO, or (ii) 1.0 μM PKRi; n = 4–6 per treatment. After 21-days media was removed and cells fixed and stained with Alizarin Red S for 5 min. Colour was removed and absorbance read at 540 nm using a plate reader. Significant differences were detected by Student's 2-sample test **p<0.01, ***p<0.001. Supplementary figure 1
