# Supplementary material for: Interferon-gamma modulates articular chondrocyte and osteoblast metabolism through protein kinase R-independent and dependent mechanisms
Source: Biochem Biophys Rep. 2022 Sep 7;32:101323. doi: 10.1016/j.bbrep.2022.101323 (PMC9464860; doi:10.1016/j.bbrep.2022.101323)
Supplement: Supplementary table 1 — Quantitative qPCR primers. [file mmc1.doc]

**Supplementary Table 1. Quantitative PCR primers**

| **Gene** | **Cross-reactivity** | **Strand** | **Sequence** | **Reference/**  **Accession No./**  **Source** |
| --- | --- | --- | --- | --- |
| ***18s*** | Bovine/mouse/  human | Forward  Reverse | 5’-GCAATTATTCCCCATGAACG-3’  5’-GGCCTCACTAAACCATCCAA-3’ |  |
| ***PP1A*** | Bovine/mouse/  human | Forward  Reverse | 5’-GGTGGTGACTTCACACGCCATAATG-3’  5’-CTTGCCATCCAACCACTCAGTCTTG-3’ | NM_178320 |
| ***YWHAZ*** | Bovine/mouse/  human | Forward  Reverse | 5’-CTGAGGTTGCAGCTGGTGATGACA-3’  5’-AGCAGGCTTTCTCAGGGGAGTTCA-3’ | NM_174814 |
| ***β-actin*** | Mouse/  human | Forward  Reverse | 5’-GATCAAGATCATTGCTCCTCCTG-3’  5’-AGGGTGTAAAACGCAGCTCA-3’ | NM_007393.5  NM_001101.5 |
| ***PKR*** | Bovine | Forward  Reverse | 5'-CTGGTCGTTCACCATGTT-3'  5'-CTCAATGGGTGGTCCTTC-3' | NM_178109 |
| ***Stat1*** | Bovine | Forward  Reverse | 5’-ATGATGGGTGTATCGTGGGC-3’  5’-TCACCTCCATTTTGGGACCG-3’ | NM_001077900 |
| ***TNFa*** | Bovine | Forward  Reverse | 5’-CATCCTGTCTGCCATCAAGA-3’  5’-GGCGATGATCCCAAAGTAGA-3’ | NM173966 |
| ***Il6*** | Bovine | Forward  Reverse | 5’-ACGAAAGAGAGCTCCATCTGC-3’  5’-AATGGAGTGAAGGCGCTTGT-3’ | NM_173923 |
| ***MMP13*** | Bovine | Forward  Reverse | 5’-CCCTTGATGCCATAACCAGT-3’  5’-GCCCAAAATTTTCTGCCTCT-3’ |  |
| ***ADAMTS4*** | Bovine | Forward  Reverse | 5’-CTCCATGACAACTCGAAGCA-3’  5’-CTAGGAGACAGTGCCCGAAG-3’ |  |
| ***PKR*** | Mouse | Forward  Reverse | 5’- ATGCACGGAGTAGCCATTACG-3’  5’- TGACAATCCACCTTGTTTTCGT-3’ | Primer Bank  NM_011163 |
| ***Stat1*** | Mouse | Forward  Reverse | 5’-GCCTCTCATTGTCACCGAAGAAC-3’  5’-TGGCTGACGTTGGAGATCACCA-3’ | NM_001357627 |
| ***TNFa*** | Mouse | Forward  Reverse | 5’- CCCTCACACTCAGATCATCTTCT-3’  5’- GCTACGACGTGGGCTACAG-3’ | Primer bank |
| ***Il6*** | Mouse | Forward  Reverse | 5’-tagtccttcctaccccaatttcc-3’  5’-TTGGTCCTTAGCCACTCCTTC-3’ | Primer bank  NM_031168 |
| ***Runx2*** | Mouse | Forward  Reverse | 5’-GACGAGGCAAGAGTTTCACC-3’  5’-GTCTGTGCCTTCTTGGTTCC-3’ | NM_009820 |
| ***Opg*** | Mouse | Forward  Reverse | 5’-GAGTGTGAGGAAGGGCGTTAC-3’  5’-GCAAACTGTGTTTCGCTCTG-3’ | NM_008764 |
| ***OCN*** | Mouse | Forward  Reverse | 5’- ccgcctacaaacgcatctat-3’  5’- ttttggagctgctgtgacat-3’ | NM_007541.2 |
| ***Smpd3*** | Mouse/Human | Forward  Reverse | 5’-ACACGACCCCCTTTCCTAATA-3’  5’-GGCGCTTCTCATAGGTGGTG-3’ | NM_021491 |
| ***Phospho1*** | Mouse | Forward  Reverse | 5’-TTCTCATTTCGGATGCCA-3’  5’-TGAGGATGCGGCGGAAT-3’ | NM_153104 |
| ***Alp*** | Mouse | Forward  Reverse | 5’-GCTGGCCCTTGACCCCTCCA-3’  5’-ATCCGGAGGGCCACCTCCAC-3’ | NM_007431 |
| ***Col1a1*** | Mouse | Forward  Reverse | 5’-ACTGCCCTCCTGACGCATGG-3’  5’-TCGCACACAGCCGTCCCATT-3’ | NM_007742.3 |
| ***PKR*** | Human | Forward  Reverse | 5’-GAAGTGGACCTCTACGCTTTGG-3’  5’-TGATGCCATCCCGTAGGTCTGT-3’ | NM_002759  Origene |
| ***STAT1*** | Human | Forward  Reverse | 5’-ATGGCAGTCTGGCGGCTGAATT-3’  5’-CCAAACCAGGCTGGCACAATTG-3’ | NM_007315  Origene |
| ***TNFA*** | Human | Forward  Reverse | 5’-CTCTTCTGCCTGCTGCACTTTG-3’  5’-ATGGGCTACAGGCTTGTCACTC-3’ | NM_000594.4  Origene |
| ***IL6*** | Human | Forward  Reverse | 5’-AGACAGCCACTCACCTCTTCAG-3’  5’-TTCTGCCAGTGCCTCTTTGCTG-3’ | NM_000600.5  Origene |
| ***RUNX2*** | Human | Forward  Reverse | 5’-TTCCCGAGGTCCATCTACTG-3’  5’-GTGGACGAGGCAAGAGTTTG-3’ | NM_001024630.4 |
| ***OPG*** | Human | Forward  Reverse | 5’-GAGATAGAGTTCTGCTTGAAACA-3’  5’-CCATCTGGACATCTTTTGCAAA-3’ | NM_002546.4 |
| ***OCN*** | Human | Forward  Reverse | 5’-CTTTGTGTCCAAGCAGGAGG-3’  5’- CTGAAAGCCGATGTGGTCAG-3’ | NM_199173.6 |
| ***PHOSPHO1*** | Human | Forward  Reverse | 5’-CCCTTCCCCACTTCTTACAC-3’  5’-AGAAACTGGAAAACAGCCAC-3’ | NM_178500.4 |
| ***ALP*** | Human | Forward  Reverse | 5’-GGCTGGAGATGGACAAGTTC-3’  5’-CCTTCACCCCACACAGGTAG-3’ | NM_000478 |
| ***COL1A1*** | Human | Forward  Reverse | 5’- CTCCTGACGCACGGCC-3’  5’- CCGTTCTGTACGCAGGTGATT -3’ | NM_000088.4 |

1. Frye, S.R., et al., *cDNA microarray analysis of endothelial cells subjected to cyclic mechanical strain: importance of motion control.* Physiol Genomics, 2005. **21**(1): p. 124-30.

2. Anstaett, O.L., et al., *Validation of endogenous reference genes for RT-qPCR normalisation in bovine lymphoid cells (BL-3) infected with Bovine Viral Diarrhoea Virus (BVDV).* Veterinary immunology and immunopathology, 2010. **137**(3-4): p. 201-7.

3. Yamane, D., et al., *The double-stranded RNA-induced apoptosis pathway is involved in the cytopathogenicity of cytopathogenic Bovine viral diarrhea virus.* J Gen Virol, 2006. **87**(Pt 10): p. 2961-2970.

4. Blain, E.J., A.Y. Ali, and V.C. Duance, *Boswellia frereana (frankincense) suppresses cytokine-induced matrix metalloproteinase expression and production of pro-inflammatory molecules in articular cartilage.* Phytother Res, 2010. **24**(6): p. 905-12.

5. Boukhechba, F., et al., *Human primary osteocyte differentiation in a 3D culture system.* J Bone Miner Res, 2009. **24**(11): p. 1927-35.
