# Supplementary material for: Interferon-gamma modulates articular chondrocyte and osteoblast metabolism through protein kinase R-independent and dependent mechanisms
Source: Biochem Biophys Rep. 2022 Sep 7;32:101323. doi: 10.1016/j.bbrep.2022.101323 (PMC9464860; doi:10.1016/j.bbrep.2022.101323)
Supplement: Supplementary table 2 — The effect of PKRi on basal gene expression was determined in (A) bovine chondrocytes (b) murine MC3T3-E1 cells, and (C) human primary osteoblasts. Cells were treated with 0.002% DMSO, or 1.0 μM PKRi (n = 3–6 per treatment) and RNA extracted from cells prior to analysis by quantitative PCR. Data are presented as fold change relative to control cells calculated using the ΔΔCT method (±S.E.M). Significant differences were detected by (A) Mann Whitney test or (B and C) Student's 2-sample test. [file mmc2.docx]

**Supplementary Table 2: The effect of PKRi on basal gene expression**

(A)

| Chondrocytes | Control | PKRi | P value |
| --- | --- | --- | --- |
| *Mmp13* | 1.024(0.07) | 0.303(0.05) | 0.001 |
| *Adamts4* | 1.036(0.11) | 0.642(0.07) | 0.007 |
|  |  |  |  |

(B)

| MC3T3-E1 | Control | PKRi | P value |
| --- | --- | --- | --- |
| *Il6* | 1.013(0.18) | 0.599(0.04) | 0.003 |
| *Runx2* | 1.033(0.26) | 0.520(0.18) | 0.008 |
| *Opg* | 0.955(0.12) | 0.511(0.13) | 0.002 |
| *Ocn* | 1.011(0.16) | 2.203(0.35) | 0.008 |
| *Smpd3* | 1.060(0.13) | 0.680(0.18) | 0.052 |
| *Phospho1* | 1.018(0.21) | 2.224(0.60) | 0.031 |
| *Alp* | 1.025(0.10) | 3.986(0.52) | 0.001 |
|  |  |  |  |

(C)

| Human osteoblasts | Control | PKRi | P value |
| --- | --- | --- | --- |
| *PKR* | 1.022(0.23) | 0.348(0.172) | 0.005 |
| *STAT1* | 1.069(0.43) | 0.405(0.23) | 0.054 |
| *RUNX2* | 1.048(0.20) | 0.173(0.08) | 0.028 |
| *OPG* | 1.055(0.18) | 0.580(0.12) | 0.079 |
| *COL1* | 1.035(0.16) | 0.588(0.08) | 0.072 |
|  |  |  |  |

**Supplementary table 2.** The effect of PKRi on basal gene expression was determined in (A) bovine chondrocytes (b) murine MC3T3-E1 cells, and (C) human primary osteoblasts. Cells were treated with 0.002% DMSO, or 1.0µM PKRi (n = 3-6 per treatment) and RNA extracted from cells prior to analysis by quantitative PCR. Data are presented as fold change relative to control cells calculated using the ∆∆CT method (±S.E.M). Significant differences were detected by (A) Mann Whitney test or (B and C) Student’s 2-sample test.
