# Supplementary figures and images for: Interferon-gamma modulates articular chondrocyte and osteoblast metabolism through protein kinase R-independent and dependent mechanisms
Source: Biochem Biophys Rep. 2022 Sep 7;32:101323. doi: 10.1016/j.bbrep.2022.101323 (PMC9464860; doi:10.1016/j.bbrep.2022.101323)

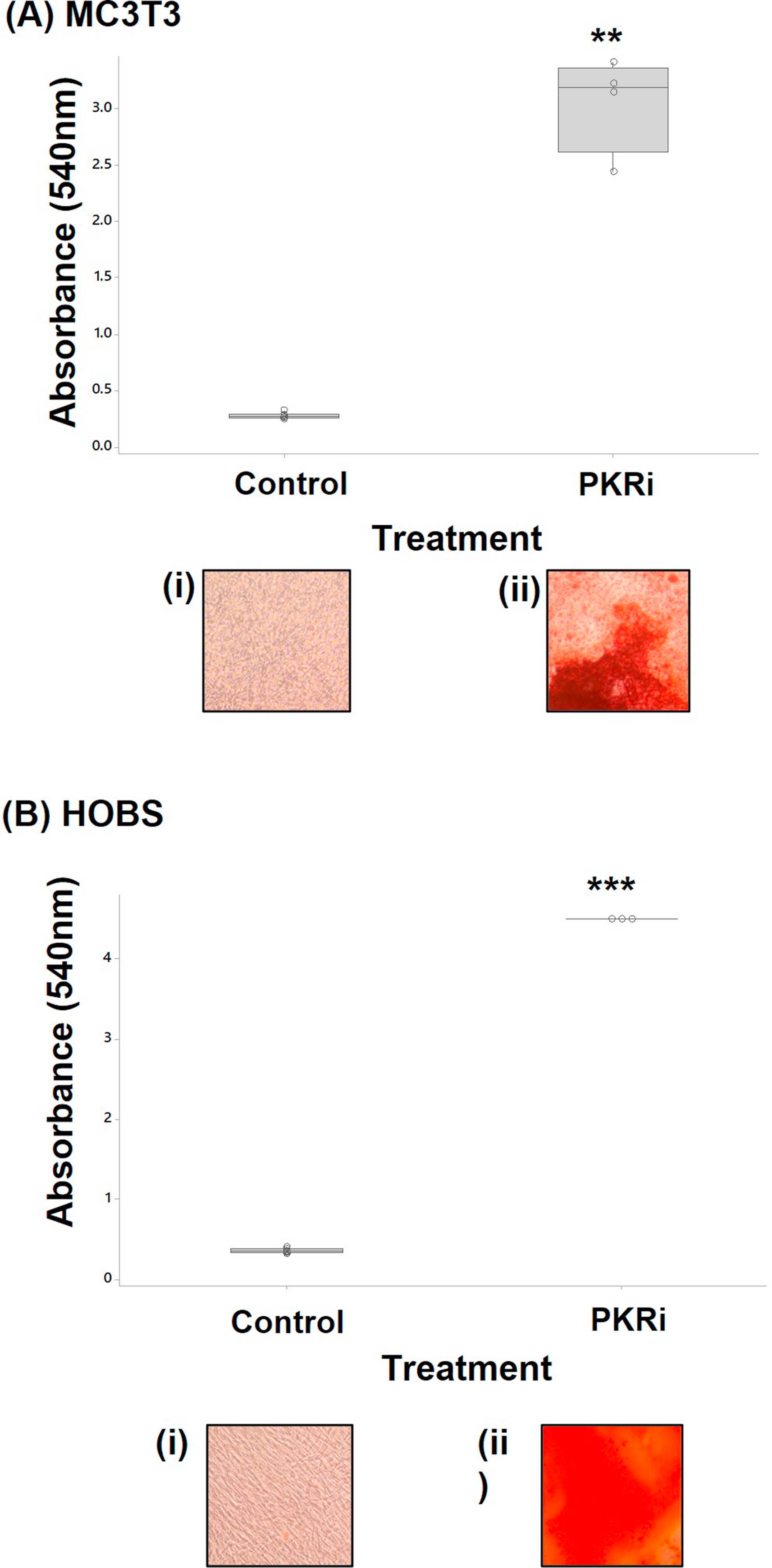

Supplement: Supplementary figure 1 — The effect of PKRi on basal mineralisation in (A) murine MC3T3-E1 and (B) human primary osteoblast cells. Cells were cultured under mineralising conditions for 21-days and treated with (i) 0.002% DMSO, or (ii) 1.0 μM PKRi; n = 4–6 per treatment. After 21-days media was removed and cells fixed and stained with Alizarin Red S for 5 min. Colour was removed and absorbance read at 540 nm using a plate reader. Significant differences were detected by Student's 2-sample test **p<0.01, ***p<0.001. [file mmcfigs1.jpg]
